# Process times of severely injured patients in the emergency room are associated with patient volume: a registry-based analysis

**DOI:** 10.1007/s00068-022-01987-y

**Published:** 2022-05-11

**Authors:** Rolf Lefering, Christian Waydhas, TraumaRegister DGU

**Affiliations:** 1 Institute for Research in Operative Medicine, University Witten/Herdecke, Ostmerheimer Strasse 200, (Building 38), 51109 Cologne, Germany; 2 Department of Surgery, BG University Hospital Bergmannsheil, Bochum, Germany; 3 Medical Faculty, University Duisburg-Essen, University Hospital, Essen, Germany

**Keywords:** Trauma, Injuries, Registry, Patient volume, Emergency room

## Abstract

**Purpose:**

Hospitals involved in the care of severely injured patients treat a varying number of such cases per year. Large hospitals were expected to show a better performance regarding process times in the emergency room. The present investigation analyzed whether this assumption was true, based on a large national trauma registry.

**Methods:**

A total of 129,193 severely injured patients admitted primarily to one of 675 German hospitals and documented in the TraumaRegister DGU^®^ were considered for this analysis. The analysis covered a 5 years time period (2013–2017). Hospitals were grouped by their average number of annually treated severe trauma patients into five categories ranging from ‘less than 10 patients’ to ‘100 or more’. The following process times were compared: pre-hospital time; time from admission to diagnostic procedures (sonography, X-ray, computed tomography), time from admission to selected emergency interventions and time in the emergency room.

**Results:**

Seventy-eight high volume hospitals treated 45% of all patients, while 30% of hospitals treated less than ten cases per year. Injury severity and mortality increased with volume per year. Whole-body computed tomography (WB-CT) was used less frequently in small hospitals (53%) as compared to the large ones (83%). The average time to WB-CT fell from 28 min. in small hospitals to 19 min. in high volume hospitals. There was a linear trend to shorter performance times for all diagnostic procedures (sonography, X-ray, WB-CT) when the annual volume increased. A similar trend was observed for time to blood transfusion (58 min versus 44 min). The median time in the emergency room fell from 74 min to 53 min, but there was no clear trend for the time to the first emergency surgery. Due to longer travel times, prehospital time was about 10 min higher in patients admitted to high volume hospitals compared to patients admitted to smaller local hospitals.

**Conclusion:**

Process times in the emergency room decreased consistently with an increase of patient volume per year. This decrease, however, was associated with a longer prehospital time.

## Introduction

It is a commonplace that with more practice and experience performance will improve. This holds also true in medicine. Surgeons know that there is a learning curve if they start a new operation technique. For the care of severely injured trauma patients there are, however, conflicting results. While some studies did not observe such a relationship [1–4], others reported a positive correlation of patient volume per trauma center with mortality and other outcome measures [5–8].

Up to now, such analyses investigating the effect of patient volume in trauma care mainly came from North America. Particularly for trauma systems with smaller sized trauma centers, as in Germany, such investigations were missing. In Germany it has been shown, however, that mortality rates differ between large and small hospitals but the standardized mortality ratio (observed vs. predicted mortality) is similar [9].

Nevertheless, this assumption led to the inclusion of an Advanced Trauma Life Support (ATLS) training for the emergency room team members into the list of criteria required to become a certified trauma center in Germany and other countries [10]. But such training sessions could only be a starting point. Knowledge and skills need permanent training and practice. However, not all hospitals could provide such a condition because of too few trauma patients.

There are nearly 700 German hospitals certified as a trauma centers. Three levels were defined: local (level 3), regional (level 2), and supra-regional (level 1) trauma centers, based on the availability of resources and, for level 1 and 2 centers, also a minimum number of severe trauma cases per year. These criteria are checked every 3 years at an on-site audit visit.

Annually, there are about 18,000 severe trauma patients in Germany, defined as Injury Severity Score (ISS) ≥ 16 [11]. About 33,000 trauma patients with maximum Abbreviated Injury Scale (AIS) severity ≥ 3, or with need for intensive care, are documented per year in the TraumaRegister DGU^®^ (TR-DGU), including the above mentioned severe trauma cases. These cases were not evenly distributed among the 700 trauma centers. The majority of cases were treated in about 100 level 1 trauma centers. But a considerable number of trauma cases were transported to a small level 3 center. Such centers often see less than ten cases per year, which means, less than one patient per month.

The present analysis does not aim to answer the question whether the outcome of severely injured trauma patient treated in a small hospital is worse than in a large trauma center. It is rather the aim of this analysis to describe the effect of low, medium, and high patient volume on the initial process times in the emergency room. Although everyone assumes that such a training effect exists, there is only scarce knowledge about the amount of this effect, or the critical number of cases per year where this effect becomes important. The results could thus be an empirical contribution to the discussion about the appropriate target hospital for severely injured patients.

## Methods

### TraumaRegister DGU^®^

The TraumaRegister DGU^®^ (TR-DGU) of the German Trauma Society (Deutsche Gesellschaft für Unfallchirurgie, DGU) was founded in 1993. The aim of this multi-centre database is a pseudonymised and standardised documentation of severely injured patients.

Data are collected prospectively in four consecutive time phases from the site of the accident until discharge from hospital: (A) Pre-hospital phase, (B) Emergency room and initial surgery, (C) Intensive care unit and (D) Discharge. The documentation includes detailed information on demographics, injury pattern, comorbidities, pre- and in-hospital management, course on the intensive care unit (ICU), relevant laboratory findings including data on transfusion, and outcome of each individual. The inclusion criterion is admission to hospital via the emergency room (trauma team activation) with subsequent intensive or intermediate care. Patients who reached the hospital with vital signs but died before admission to ICU were included as well.

The infrastructure for documentation, data management, and data analysis is provided by AUC—Academy for Trauma Surgery (AUC—Akademie der Unfallchirurgie GmbH), a company affiliated to the German Trauma Society. The scientific leadership is provided by the Committee on Emergency Medicine, Intensive Care and Trauma Management (Sektion NIS) of the German Trauma Society. The participating hospitals submit their data pseudonymised into a central database via a web-based application. Scientific data analysis is approved according to a peer review procedure laid down in the publication guideline of TR-DGU.

The participating hospitals are primarily located in Germany (90%), but a rising number of hospitals of other countries contribute data as well (at the moment from Austria, Belgium, Finland, Luxembourg, Slovenia, Switzerland, The Netherlands, and the United Arab Emirates). Currently, approx. 33,000 cases from over 650 hospitals are entered into the database per year. Participation in TR-DGU is voluntary. For hospitals associated with TraumaNetzwerk DGU^®^, however, the entry of at least a basic data set is obligatory for reasons of quality assurance.

This study was conducted according to the publication guideline of the TR-DGU and registered as project number 2019-007.

### Patients and hospitals

Patients documented in TR-DGU were selected from a 5-year period (January 2013–December 2017). Only cases admitted directly from the scene to a German trauma center qualified for analysis. Patients with minor injuries were excluded: all cases with maximum Abbreviated Injury Scale (MAIS) severity = 1, and all surviving cases with MAIS = 2 without intensive care. These criteria left 129,193 severely injured patients from 675 German hospitals for analysis.

Hospitals were then grouped according to their average annual number of severely injured patients into one of five subgroups: 1–9 cases per year (< 10); 10–19 cases per year (10 +); 20–39 cases per year (20 +); 40–99 cases per year (40 +), and 100 cases or more per year (100 +). Years without documented cases, or years with incomplete documentation (< 40% of the 5-year annual average) were not considered when the respective number of patients per year for categorization was determined.

Prognosis of hospital mortality was estimated using the Revised Injury Severity Classification score, version 2 (RISC 2) which was developed and validated in TR-DGU patients [12].

### Process times

The prehospital time was the interval from the accident (measured or estimated) until first hospital admission. Date and time of hospital admission is a mandatory data item. Date and time of injury was available in 78% of cases. Process times in the emergency room (minutes) were counted from hospital arrival to the start of the respective diagnostic or therapeutic intervention. Availability of time data for these procedures ranged from 95 to 97%.

If the calculated intervention times in the emergency room were > 4 h, these interventions were disregarded (no acute intervention). Total times in the emergency room > 10 h were excluded as well (waiting for transfer; 0.7% of cases).

Some variables were documented only since the latest dataset revision in 2015 and thus available for a smaller number of cases only; these measurements were indicated. This affects the time for each emergency procedure and the time to blood transfusion. For eight emergency procedures (laparotomy, thoracotomy, brain decompression, embolization, laminectomy, revascularization, external stabilization of the pelvis, external stabilization of extremities) the starting times were documented separately, and thus the time since admission could be calculated.

### Statistics

Counts were presented as percentage, and continuous measures were presented as mean with standard deviation (SD), or as median with inter-quartile range (IQR) in case of skewed distributions. For selected findings both mean and median are presented since the median is not sensitive for outliers. A formal statistical testing was avoided due to the very large sample size (several thousands of patients within each subgroup) and the multiplicity of pairwise comparisons. Thus, differences of clinically relevant size (larger than 1–2 min) could also be considered as statistically significant. For selected variables, however, the 100+ subgroup was compared to the other subgroups using the Mann–Whitney *U* test. All analyses were performed using SPSS statistical software (version 24, IBM Inc., Armonk NY, USA).

## Results

Hospitals were grouped into five subgroups according to their annual number of severely injured patients admitted primarily to the emergency room of each hospital. Most patients were treated in the 78 large hospitals with more than 100 cases per year (*n* = 58,474; 45.3%). There were 203 hospitals (30%) with less than 10 cases/year. In these 203 ‘small’ hospitals 3956 patients (3.1%) were treated. Table 1 shows the demographic and injury data of the five subgroups of hospitals.

**Table 1:** Patient demographics, injury characteristics, diagnostics performed, transfers, and outcome in hospitals of varying patient volume

No. of cases per year per hospital		< 10	10 +	20 +	40 +	100 +	Total
Number (percentage) of patients	*n*	3,956 (3.1%)	7,748 (6.0%)	18,917 (14.6%)	40,062 (31.0%)	58,474 (45.3%)	129,193 (100%)
Number (percentage) of hospitals	*n*	203 (30%)	127 (19%)	134 (20%)	133 (20%)	78 (12%)	657 (100%)
Age (years)	Mean (SD)	54 (22)	53 (22)	54 (23)	52 (22)	49 (22)	51 (22)
Male gender	%	69%	68%	67%	69%	71%	70%
ISS	Mean (SD)	15 (9)	15 (9)	16 (10)	18 (11)	19 (12)	18 (12)
ISS ≥ 16	%	40%	40%	45%	51%	53%	50%
Head injury (AIS ≥ 3)	%	21%	21%	27%	32%	38%	33%
Total pre-hospital time from accident to hospital (min.)	Median (IQR)	50 (38–65)	50 (39–65)	51 (40–66)	57 (44–75)	60 (47–80)	57 (44–75)
Travel time from scene to hospital (min.)	Median (IQR)	14 (9–20)	15 (10–21)	14 (9–20)	16 (10–24)	16 (10–24)	16 (10–23)
Transportation by helicopter	%	1%	2%	3%	16%	30%	19%
Whole-body CT	%	59%	63%	68%	79%	83%	77%
Chest X-ray	%	36%	36%	31%	28%	39%	34%
Focussed abdominal sonography (FAST)	%	83%	86%	84%	84%	88%	86%
Blood transfusion (until ICU)	%	5%	4%	5%	7%	8%	7%
Patients with an emergency surgery	%	16%	16%	20%	23%	24%	23%
Early transfer out to another hospital	%	19%	14%	10%	3%	< 1%	4%
Hospital mortality^a^	%	5.9%	6.4%	7.8%	10.1%	11.9%	9.6%
Expected mortality based on RISC II^a^	%	5.9%	6.0%	7.4%	9.7%	11.3%	9.9%
Length of stay in hospital^a^ (days)	median (IQR)	9 (5–15)	9 (5–15)	10 (5–17)	11 (6–19)	12 (6–22)	11 (6–20)

*SD*   standard deviation; *IQR*   inter-quartile range

^a^Without patients transferred out early (< 48 h) to other hospitals

Patient characteristics are presented in Table 1. Patients admitted to high volume hospitals tended to be younger, were more severely injured, had more head injuries, and their hospital mortality was higher. Every fifth patient (19%) admitted to a small hospital with less than ten cases per year was transferred out to another hospital within a few hours.

**Fig. 1:**
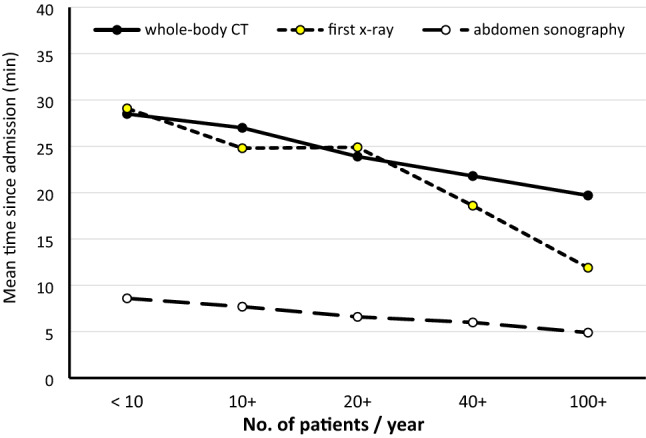
Time to diagnostic procedures. Time from admission to first diagnostic procedures in subgroups of hospitals with varying volume per year. The X-rays considered here were chest, spine, and pelvis

There were also differences in the frequency of using diagnostic procedures. While abdominal ultrasound (Focused Assessment with Sonography for Trauma, FAST) and chest X-ray showed approximately similar prevalence rates, there was a clear shift in using computed tomography (CT). The use of whole-body CT increased from 59% in the smallest hospitals to 83% in the large ones. The average times since admission needed to start these investigations are presented in Fig. 1. If time to first CT was limited to cases with GCS < 14 only (38% of all cases) average time intervals did not differ from the overall times (difference within a range of ± 1 min per hospital category).

If smallest and largest hospitals were compared, time to whole-body CT was reduced by about one third (9 min. faster in large hospitals), time to FAST was approximately halved (4 min. faster), and time to first X-ray was even more than halved (17 min. faster).

**Fig. 2:**
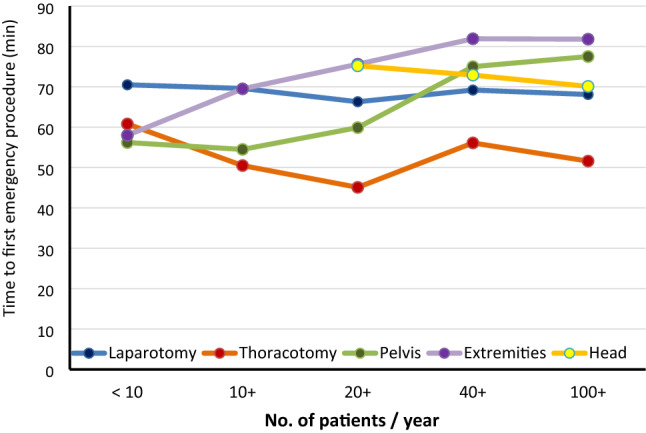
Time to emergency procedures. Mean time from admission to start of selected emergency procedures, in subgroups of hospitals with varying volume per year (subgroup results not presented if based on less than 15 cases)

For a set of eight surgical emergency procedures, time intervals were documented separately in TR-DGU. Those procedures were more frequently performed in hospitals with a higher annual volume, and thus higher injury severity. The mean time to the first procedure increased with volume: in small hospitals it took 60 min on average (SD 33), while it took 74 min (SD 27) in hospitals with the highest volume. However, this trend is not identical for all emergency interventions. Figure 2 shows that this delay is mainly caused by external stabilization of pelvis and extremities.

The median time to start blood transfusion was 35 min in large hospitals (100 + cases/year) and 47 min in small hospitals.

**Fig. 3:**
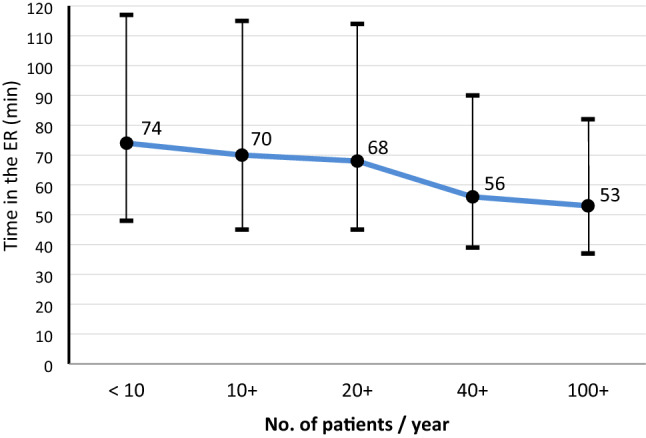
Median time in the emergency room. Median (with IQR) duration of treatment in the emergency room of all patients, in subgroups of hospitals with varying volume per year

Table 2 describes the total time in the emergency room (ER). Overall, there is a considerable and consistent decrease in length of stay in the ER with increasing volume (Table 2 and Fig. 3).

**Table 2:** Time spent in the emergency room (ER) from admission to discharge or transfer, for different discharge destinations, or death in the ER

No. of cases per year per hospital	< 10	10 +	20 +	40 +	100 +	Total
All patients treated in the ER	1440	2953	7324	14,875	22,005	48,597
	100%	100%	100%	100%	100%	100%
	93/74	90/70	92/68	78/56	73/53	79/57
	(69)	(69)	(74)	(66)	(67)	(68)
Discharged from ER to ICU	822	1,857	4,580	9,906	14,190	31,355
	57%	63%	63%	67%	65%	65%
	85 /.62	83/60	84/60	77/55	77/53	79/55
	(68)	(68)	(71)	(67)	(74)	(71)
Discharged from ER to operation room	175	404	1,316	3,371	6,366	11,632
	12%	14%	18%	23%	29%	24%
	98/75	85/69	90/68	71/56	65/52	71/55
	(81)	(62)	(72)	(53)	(51)	(56)
Discharged from ER to another hospital	310	452	844	606	146	2,358
	22%	15%	12%	4%	0.7%	5%
	116/105	127/114	125/107	129/115	119/101	125/108
	(63)	(70)	(76)	(77)	(83)	(74)
Died in the ER	21	48	116	221	381	787
	1.5%	1.6%	1.6%	1.5%	1.7%	1.6%
	39/16	44/26	49/35	44/35	44/33	44/33
	(50)	(53)	(50)	(41)	(42)	(44)

Limited to patients with time data available since dataset revision 2015; maximum time was limited to 10 h

All data presented as: no. of patients, percentage within hospital category, mean/median (standard deviation). 2465 cases with other destinations were not reported separately here but included in the total time

787 patients (1.6%) died in the ER; their median time to death was 33 min, and this time did not differ across the hospital subgroups. Another group of patients were directly transferred from the ER to another hospital. Their fraction decreased from 22% in hospitals with lowest volume to less than 1% in hospitals with highest volume. On average, they stayed about 2 h in the ER before transfer, with only minor variation across the hospitals. The largest group of patients was discharged from ER to ICU. Their length of stay in the ER only slightly decreased with increasing volume (on average 10 min faster in high volume hospitals). The largest time savings were observed in patients transferred to the operation room for surgery. Not only the percentage of cases discharged to the OR was highest in large hospitals (29%), but also the time in the ER was shortest (mean time 65 min, Table 2). This value also had the lowest SD indicating that there were less outliers with a long delay.

**Fig. 4:**
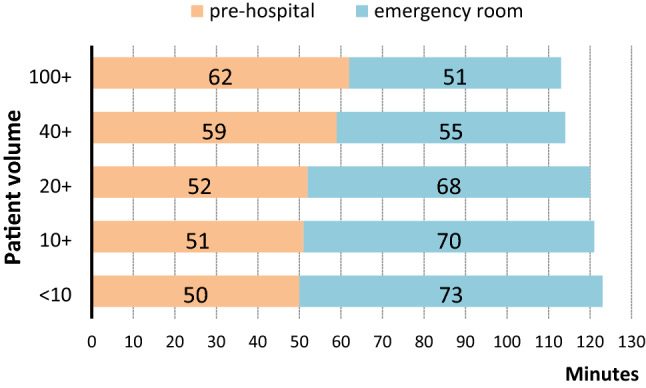
Pre-hospital time and time in the emergency room. Median prehospital time from accident until hospital admission, and median time in the emergency room, in subgroups of hospitals with varying volume per year. Only patients discharged to the intensive care unit or to the operating room were included in this figure (*n* = 42,987)

The median prehospital time from the accident to hospital admission was 9–12 min longer for large hospitals treating 40 cases/year or more (Fig. 4). There is a net time saving of about 10 min (from the accident to the end of ED treatment) for large hospitals if the time in the ED is added.

## Discussion

There is an ongoing debate whether severely injured patients should be brought to the closest hospital nearby to save time, or to directly admit such a patient to the next ‘appropriate’ trauma center with adequate resources, even with bypassing the closest hospital. Those who promote the second option argue that the additional time required for transportation will easily be compensated by a more efficient acute care in specialized centers. Some people even promote to establish ‘mega centers’ with several hundreds of severe trauma cases (ISS 16+) per year. However, too long transportation times may also result in a case selection bias excluding instable trauma victims who quickly died.

The present analysis is based on a large nation-wide trauma registration in Germany. We do not have such ‘mega centers’ here and could therefore not contribute to that discussion. However, we have a large number of hospitals (about 700) participating in the treatment of severely injured patients. And most of these hospitals only see a limited number of patients per year. This would allow comparing process times in the acute phase even if the patient volume is low.

We classified hospitals by their annual number of severely injured patients (based on a 5 years average) which is more specific than using the certified level of care. Since our documentation also includes the pre-hospital setting (times, physiology, and interventions), we were able to quantify the additional time needed to bring a patient to a high volume hospital with appropriate resources.

As expected, there is a relation between patient volume and process times. This relation is most prominent for time to first CT, first X-ray, and overall time in the ER, especially for patients transferred to the operation room. But we were not able to define an appropriate cut-off point regarding patients per year. The considered times still decreased up to the highest volume subgroup of hospitals. For very large hospitals we did not have enough data; there were only nine hospitals with > 200 patients/year. This is a clear argument that frequent training will reduce process times. Interestingly, the average time until CT-scanning was 20 min in the highest volume centers and 29 min in the lowest volume centers in our study, while the median time until CT-scanning was reported to be 82 min in a Dutch very high-volume trauma center [13]. Therefore, international comparisons with results from different health care systems have to be interpreted with caution.

Interestingly, not all emergency interventions showed this trend. The time to some acute interventions did not vary across the hospitals (e.g., laparotomy with about 70 min). Time to other interventions, like external fixation, even took longer time in large hospitals. This might be caused by prior treatment of other life-threatening injuries, or a successful stabilization and resuscitation, which reduces the urgency. Other interventions like a thoracotomy showed a tendency towards a faster performance. While it is not clear whether time savings until diagnostic laparotomy lead to improved outcome [14] a shorter time until emergency thoracotomy correlates with a reduction in mortality [7].

On the other hand, there is a discussion about the additional time needed to bring a patient directly to a large trauma center. According to our results, this difference is rather small (about 10 min), compared to the time savings in the ER. The longer pre-hospital time could be explained by the higher injury severity requiring additional interventions at scene, and the use of helicopters for transportation [15]. This is mainly because the helicopter is usually not the primary vehicle but will be dispatched on request by the ground EMS team. The pure transportation time from scene to hospital is only 2 min longer in cases admitted to large hospitals. This delay is compensated already after the first few minutes in hospital.

It was not our aim to evaluate the relationship of patient volume and outcome in this paper. Such a relationship is often assumed, and it has also been shown by several authors in trauma care [8, 16–19], while other authors could not prove such a relationship [3]. Previous analyses from TR-DGU in ISS 16+ patients (without transfers) suggested an adequate severity-adjusted outcome if more than 40 patients were treated annually [20]. In the present study, however, observed and expected mortality were nearly identical in all subgroups, if transfers were excluded. But we could not answer the question whether initial stabilization in a small hospital with subsequent transfer, or direct admission to a large trauma center is associated with a superior outcome. This is because our registry system does not allow matching treatment phases in different hospitals with sufficient precision. But if only process times were considered then longer travel times to a large trauma center were more than balanced after the initial shock room phase. This requires, however, a dense distribution of large trauma centers, as given in Germany.

Process times are frequently used as quality indicators (QI). A recently performed expert rating of 40 QI favored such process times at least for whole-body CT and several emergency interventions [21]. The subsequent empirical validation of these QI using TR-DGU data found a stronger association of event-related QI rather than process times [22]. In other words, it is more important that a certain intervention is performed, rather than it is performed 10 min earlier or later.

Our investigation bears all limitations of registry studies: there is no verification of correctness, and missing values were not queried. The exclusion of cases with missing or implausible time data may thus induce a bias. However, the online documentation software has a large number of plausibility checks implemented. The TR-DGU is well-known in Germany, and runs already for 25 years, and process times in the ER have been collected since the beginning. Amalgamation of treatment phases from different hospitals could not be performed due to a missing unique case identifier in Germany. This limits the outcome analysis of transferred cases.

Furthermore, the present results are valid for the German trauma system only which has some specific characteristics: nearly all trauma victims were seen by a physician at the scene who decides about the transport destination. Traditionally, the number of hospitals engaged in trauma care is high which leads to a large number of hospitals involved but with low patient volume per hospital. On the other hand, all participating hospitals were certified by the German Trauma Society and have shown to fulfill the requirements as laid down in the national Whitebook for the Medical Care of the Severely Injured [23]. Among other criteria they provide 24 h availability of CT, defined trauma teams, ATLS training for at least 50% of physicians in the trauma teams, etc. Therefore, a high level of expertise is present even in certified lower volume hospitals. Furthermore, population density is generally high in Germany so that a hospital could be reached within a few minutes, in most areas. An extended air rescue system with 89 helicopters further helps to reduce the pre-hospital time.

## Conclusion

For severely injured patients, there is a clear and linear association of patient volume and process times in the emergency room, including several diagnostic procedures and the overall time in the ER. Up to 20 min could be saved per patient in large hospitals with more than 100 severe trauma cases per year. On the other hand, the increase in pre-hospital transportation time is less distinct.

## Data Availability

According to the guidelines of the German Trauma Society (DGU) and the Akademie der Unfallchirurgie (AUC GmbH) who runs the registry, data from TR-DGU are not publicly available. However, applications for analyses could be forwarded to AUC. Requests for data details regarding the present project will be answered by the corresponding author.

## References

[1] London JA, Battistella FD (2003). Is there a relationship between trauma center volume and mortality?. J Trauma.

[2] Glance LG, Osler TM, Dick A, Mukamel D (2004). The relation between trauma center outcome and volume in the National Trauma Databank. J Trauma.

[3] Calland JF, Stukenborg GJ (2016). Trauma centre patient volume and inpatient mortality risk reconsidered. Injury.

[4] Tsai SHL, Goyal A, Alvi MA, Kerezoudis P, Yolcu YU, Wahood W, Habermann EB, Burns TC, Bydon M (2020). Hospital volume-outcome relationship in severe traumatic brain injury: stratified analysis by level of trauma center. J Neurosurg.

[5] Stawicki SP, Habeeb K, Martin ND, O'Mara MS, Cipolla J, Evans DC (2019). A seven-center examination of the relationship between monthly volume and mortality in trauma: a hypothesis-generating study. Europ J Trauma Emerg Surg.

[6] Elkbuli A, Eily A, Hai S, McKenney M, Morejon O (2018). The impact of trauma center patient volume on observed/expected mortality: does size matter?. Am Surg.

[7] Dumas RP, Seamon MJ, Smith BP, Yang W, Cannon JW, Schwab CW (2018). The epidemiology of emergency department thoracotomy in a statewide trauma system: does center volume matter?. J Trauma Acute Care Surg.

[8] Brown JB, Rosengart MR, Kahn JM, Mohan D, Zuckerbraun BS, Billiar TR, Peitzman AB, Angus DC, Sperry JL (2017). Impact of volume change over time on trauma mortality in the United States. Ann Surg.

[9] TraumaRegister DGU—Annual report 2019. http://www.traumaregister-dgu.de/index.php?id=1385. Accessed 6 Jan 2020

[10] Ruchholtz S, Lefering R, Lewan U, Debus F, Mand C, Siebert H, Kühne CA (2014). Implementation of a nationwide trauma network for the care of severely injured patients. J Trauma Acute Care Surg.

[11] Debus F, Lefering R, Kühne CA, Frink M, Mand C, Bücking B, Ruchholtz S (2015). Number of severely injured patients in Germany. Dt Ärzteblatt Int.

[12] Lefering R, Huber-Wagner S, Nienaber U, Maegele M, Bouillon B (2014). Update of the trauma risk adjustment model of the TraumaRegister DGU: the Revised Injury Severity Classification, version II. Crit Care.

[13] Fung Jin Kon PH, van Geene AR, Linnau KF, Jurkovich GJ, Ponsen KJ, Goslings JC (2009). Time factors associated with CT scan usage in trauma patients. Eur J Radiol.

[14] Lewis PR, Badiee J, Sise MJ, Calvo RY, Brill JB, Wallace JD (2017). "Delay to operating room" fails to identify adverse outcomes at a Level I trauma center. J Trauma Acute Care Surg.

[15] Wyen H, Lefering R, Maegele M, Brockamp T, Wafaisade A, Wutzler S, Walcher F, Marzi I (2013). The golden hour of shock-how time is running out pre-hospital time intervals in Germany: a multivariate analysis of 15,103 patients from the Traumaregister DGU. Emerg Med J.

[16] Hentschker C, Mennicken R (2018). The volume-outcome relationship revisited: practice indeed makes perfect. Health Serv Res.

[17] Freeman J, Nichol J, Turner J (2006). Does size matter? The relationship between volume and outcome in the care of major trauma. J Health Serv Res Policy.

[18] Bell TM, Boustany KC, Jenkins PC, Zarzaur BL (2015). The relationship between trauma center volume and in-hospital outcomes. J Surg Res.

[19] Aoki M, Abe T, Saitoh D, Hagiwara S, Oshima K (2020). Severe trauma patient volume was associated with decreased mortality. Europ J Trauma Emerg Med.

[20] Zacher M, Kanz KG, Hanschen M, Häberle S, van Griensven M, Lefering R, Bühren V, Biberthaler P, Huber-Wagner S, TraumaRegister DGU (2015). Association between volume of injured patients and mortality in German trauma hospitals. Br J Surg.

[21] Bieler D, Hörster A, Lefering R, Franke A, Waydhas C, Huber-Wagner S, Baacke M, Paffrath T, Wnent J, Volland R, Jackisch B, Walcher F, Kulla M (2020). Evaluation of new quality indicators for the TraumaRegister DGU^®^ using systematic QUALIFY methodology. Eur J Trauma Emerg Surg.

[22] Hörster A, Kulla M, Bieler D, Lefering R (2019). Empirische Überprüfung der Qualitätsindikatoren für Schwerverletzte im TraumaRegister DGU^®^. Unfallchirurg.

[23] German Society for Trauma Surgery (Ed.) (2012) Whitebook Medical Care of the Severely Injured. 2nd revised and updated edition. Berlin 2012. http://www.dgu-online.de. Accessed 12 June 2020

